# Impact of hormone receptor status on patterns of recurrence and clinical outcomes among patients with human epidermal growth factor-2-positive breast cancer in the National Comprehensive Cancer Network: a prospective cohort study

**DOI:** 10.1186/bcr3324

**Published:** 2012-10-01

**Authors:** Ines Vaz-Luis, Rebecca A Ottesen, Melissa E Hughes, P Kelly Marcom, Beverly Moy, Hope S Rugo, Richard L Theriault, John Wilson, Joyce C Niland, Jane C Weeks, Nancy U Lin

**Affiliations:** 1 Department of Medical Oncology, Dana-Farber Cancer Institute, Boston, MA, USA; 2 Clinical and Translational Oncology Research Unit, Instituto de Medicina Molecular, Lisbon, Portugal; 3 Department of Information Sciences, City of Hope Comprehensive Cancer Center, Duarte, CA, USA; 4 Department of Medical Oncology, Duke University Medical Center, Durham, NC, USA; 5 Department of Medicine, Massachusetts General Hospital, Boston, MA, USA; 6 Department of Medicine, San Francisco Helen Diller Family Comprehensive Cancer Center, San Francisco, CA, USA; 7 Department of Breast Medical Oncology, University of Texas M. D. Anderson Cancer Center, Houston, TX, USA; 8 Department of Internal Medicine, Arthur G. James Cancer Hospital, Ohio State University, Columbus, OH, US

## Abstract

**Introduction:**

In gene expression experiments, hormone receptor (HR)-positive/human epidermal growth factor-2 (HER2)-positive tumors generally cluster within the luminal B subset; whereas HR-negative/HER2-positive tumors reside in the HER2-enriched subset. We investigated whether the clinical behavior of HER2-positive tumors differs by HR status.

**Methods:**

We evaluated 3,394 patients who presented to National Comprehensive Cancer Network (NCCN) centers with stage I to III HER2-positive breast cancer between 2000 and 2007. Tumors were grouped as HR-positive/HER2-positive (HR+/HER2+) or HR-negative/HER2-positive (HR-/HER2+). Chi-square, logistic regression and Cox hazard proportional regression were used to compare groups.

**Results:**

Median follow-up was four years. Patients with HR-/HER2+ tumors (n = 1,379, 41% of total) were more likely than those with HR+/HER-2+ disease (n = 2,015, 59% of total) to present with high histologic grade and higher stages (*P *<0.001). Recurrences were recorded for 458 patients. HR-/HER2+ patients were less likely to experience first recurrence in bone (univariate Odds Ratio (OR) = 0.53, 95% Confidence Interval (CI): 0.34 to 0.82, *P *= 0.005) and more likely to recur in brain (univariate OR = 1.75, 95% CI: 1.05 to 2.93, *P *= 0.033). A lower risk of recurrence in bone persisted after adjusting for age, stage and adjuvant trastuzumab therapy (OR = 0.53, 95% CI: 0.34 to 0.83, *P *= 0.005) and when first and subsequent sites of recurrence were both considered (multivariable OR = 0.55, 95% CI: 0.37 to 0.80, *P *= 0.002).

As compared with patients with HR+/HER2+ disease, those with HR-/HER2+ disease had significantly increased hazard of early, but not late, death (hazard ratio of death zero to two years after diagnosis = 1.92, 95% CI: 1.28 to 2.86, *P *= 0.002, hazard ratio of death two to five years after diagnosis = 1.55, 95% CI: 1.19 to 2.00, *P *= 0.001; hazard ratio of death more than five years after diagnosis = 0.81, 95% CI: 0.55 to 1.19, *P *= 0.285, adjusting for age, race/ethnicity, stage at diagnosis, grade and year of diagnosis).

**Conclusions:**

Presenting features, patterns of recurrence and survival of HER2-positive breast cancer differed by HR status. These differences should be further explored and integrated in the design of clinical trials.

## Introduction

Breast cancer is a heterogeneous disease, with substantial genotypic and phenotypic diversity [1]. In gene expression experiments, hormone receptor (HR) segregates tumors before human epidermal growth factor 2 (HER2), suggesting that HR status is the most important discriminator of breast cancers. HR-positive is used to categorize breast tumors in two major groups [1-3]: luminal A and luminal B and HR-negative cancers include normal-like, HER2 enriched, basal and claudin-low subtypes [1,4-6]. Most of the clinically HR-positive/HER2-positive (HR+/HER2+) cancers tend to fall in the luminal B subtype, and most of HR-negative/HER2-positive (HR-/HER2+) in the HER2 enriched subtype [7].

In clinical practice, patients with HR+/HER2+ tumors routinely receive endocrine therapy. In some cases, the threshold to consider chemotherapy and trastuzumab is slightly higher than in patients with HR-/HER2+ tumors. However, despite their molecular differences, HER2-positive tumors are often treated as a single entity, and trials for HER2-positive disease typically include patients irrespective of HR status.

Increasingly, some differences in clinical outcomes, timing and patterns of dissemination within HER2-positive disease according to HR have been reported [8-16]. However, adjuvant trials have frequently reported only the sites of initial, but not subsequent, recurrence. Because of selection bias, studies of patients with metastatic disease are not ideal for examining sites and timing of recurrence associated with HR status in HER-2-positive disease overall.

Since 1997, the National Comprehensive Cancer Network (NCCN) Breast Cancer Outcomes Database has collected data on women with newly diagnosed breast cancer presenting to many of its member institutions across the United States [17]. The large size of the cohort allows for investigation of the impact of HR within HER2-positive breast tumors, with potentially less bias than relying on clinical trial data alone. We aimed to describe clinicopathological features, patterns of recurrence and clinical outcomes of HER2-positive breast cancer according to HR status.

## Methods

### Data source

Data are collected prospectively within the NCCN Database mainly through review of medical records and institutional tumor registries. Vital status and cause of death are determined from medical records and confirmed using the Social Security Death Index and the National Death Index (NDI). Data are subjected to rigorous quality assurance [18]. Institutional review boards from each center approved the study. At centers where a signed informed consent was required, only patients who provided consent were included; elsewhere, the institutional review boards grant a waiver of signed informed consent.

### Patient selection

Patients were included in the analytic group if they presented with newly diagnosed, stage I to III, unilateral, HER2-positive invasive breast cancer between January 2000 and December 2007, at 1 of 13 NCCN institutions: Arthur G. James Cancer Hospital at Ohio State University (Columbus, OH), City of Hope Comprehensive Cancer Center (Duarte, CA), Dana-Farber Cancer Institute (Boston, MA), Duke University Medical Center (Durham, NC), Fox Chase Cancer Center (Philadelphia, PA), H. Lee Moffitt Cancer Center (Tampa, FL), Massachusetts General Hospital (Boston, MA), Robert H. Lurie Comprehensive Cancer Center of Northwestern University (Chicago, IL), Roswell Park Cancer Institute (Buffalo, NY), Seattle Cancer Care Alliance (Seattle, WA), Siteman Cancer Center (St. Louis, MO), The University of Texas M.D. Anderson Cancer Center (Houston, TX), and University of Michigan Comprehensive Cancer Center (Ann Arbor, MI). Of 3,395 eligible women, one had unknown HR status and was excluded, leaving an analysis sample size of 3,394 patients.

### Definition of HER2 subgroups

Tumors were grouped as HR-positive (estrogen receptor (ER) and/or progesterone receptor (PR) positive)/HER2-positive and HR-negative (ER and PR negative)/HER2-positive, using pathologic information of the primary tumor, as specified below.

### Covariates of interest

#### Tumor characteristics

The database contains information on tumor size, nodal status, grade, lymphovascular invasion (LVI), extensive intraductal component (EIC), ER and PR status and HER2 status, as abstracted from pathology reports. Stage is assigned according to the version of the American Joint Committee on Cancer (AJCC) Staging Manual applicable at the time of diagnosis. Tumor grade was categorized as high (according to histologic grade, or, if not available, by nuclear grade) or low-intermediate. For HER2 classification, we used the fluorescence *in situ *hybridization (FISH) result, if available. If only immunohistochemistry (IHC) was available, 3+, "high positive" or "positive NOS" were considered HER2-positive; while 2+, 1+, 0 or "negative" were considered HER2-negative; 6% (n = 199) of patients were "positive NOS".

#### Patient characteristics

We used the following variables collected by chart review: age at diagnosis, height and weight, method of detection, sites of breast cancer recurrence, treatment types and vital status. Body mass index (BMI) was calculated as weight(kg)/height(m)^2 ^[1] and grouped according to categories defined by the National Heart, Lung and Blood Institute as follows: <18.5 kg/m2 underweight; 18.5 to 24.9 normal; 25.0 to 29.9 overweight, and >30.0 obese.

Data on race/ethnicity, menopausal status and comorbidity score [19] came from patient surveys conducted at initial presentation to the NCCN center. Patients were considered postmenopausal if they were amenorrheic for more than six months prior to breast cancer diagnosis, were taking hormone replacement therapy or were at least 50 years of age without a documented menopausal status in their medical record or baseline patient survey.

### Statistical analyses

#### Study cohort characterization

The clinicopathological features at the time of diagnosis were tabulated by HR status and proportions were compared between groups using chi-square tests. This was performed for the overall cohort, and separately among the subgroup of patients with documented recurrence. Treatment variables also were tabulated at the time of diagnosis and at the time of recurrence by HR status.

#### Sites of recurrence analysis

Sites of recurrence were tabulated by HR status in the overall cohort, the subgroup of patients with early recurrence (defined as those diagnosed within five years of initial diagnosis) and the subgroup of patients with late recurrence (those diagnosed more than five years from initial diagnosis). In the overall cohort, univariate followed by multivariable logistic regression estimated the risk of sites of recurrence (first and first plus subsequent sites of recurrence). In the multivariable analysis, the estimated risk of recurrence was adjusted by age at diagnosis (<50, ≥50 years), stage at diagnosis (I, II, III), and adjuvant trastuzumab (yes/no).

#### Agreement between primary and metastatic samples analysis

Among patients with data from paired primary and metastatic samples, agreement (yes or no) between ER, PR and HER2 status was tabulated and inter-rater agreement between ER, PR and HER2 in primary and recurrent samples was calculated (simple kappa (K) coefficients and asymptotic standard error (ASE) were used).

#### Survival outcomes analysis

Follow-up for overall survival (OS) and breast cancer specific survival (BCSS) were defined as time in years from diagnosis to date of death or last known vital status. BCSS was determined by identifying cause of death due to breast cancer based on the International Statistical Classification of Disease codes. Cox proportional hazards regression was used to calculate OS while adjusting for covariates. Hazard ratios and their associated 95% confidence interval (95% CI) of any death, and breast cancer-specific death, are presented by HR status (using HR positive as the reference group) and also grouped by HR and adjuvant trastuzumab use. In the latter analysis, the following groups were assembled: patients with HR-positive tumors who received adjuvant trastuzumab, patients with HR-negative tumors who received adjuvant trastuzumab, patients with HR-positive tumors who did not receive adjuvant trastuzumab, and patients with HR-negative tumors who did not receive trastuzumab; the first group was used as the reference group. Cox models were adjusted for age at diagnosis (<50, ≥50 years), race (Caucasian, African American, Other, Unknown), stage at diagnosis (I, II, III), grade (Low/Intermediate, High), and year of diagnosis (2000 to 2007). The risk of death since diagnosis by HR was found to be non-proportional and, therefore, HR was modeled as a time dependent covariate. The cut-points of zero to two, two to five and more than five years were selected *a priori *because it is well known that, after diagnosis, the peak hazard of recurrence in breast cancer occurs within the first two years, decreases consistently in the interval of two to five years, and decreases even more slowly beyond five years [20]. All *P*-values presented are two-sided; a *P*-value less than 0.05 was considered significant. All statistical analyses were performed using SAS 9.2 (SAS Institute Inc., Cary, NC, USA).

## Results

### Description of the study cohort

A total of 3,394 patients were included in this analysis. Subgroup distribution was: HR+/HER2+ 59% (n = 2,015) and HR-/HER2+ 41% (n = 1,379). Median follow-up time was four years (range: 0 to 11 years). The mean age at diagnosis was 52 years. The majority (79%) of patients were Caucasian.

### Clinicopathological characteristics and patterns of care

Compared to patients with HR+/HER2+ tumors, patients with HR-/HER2+ tumors were more likely to present with higher T stage (T3 to T4, 17% vs. 10%, *P *<0.001), nodal involvement (52% vs. 45%, *P *<0.001), higher AJCC stage (Stage III, 28% vs. 18%, *P *<0.001) and higher histologic grade (81% vs. 60%, *P *<0.001). Approximately one-third of women in both groups presented on the basis of a screening mammogram. Only small absolute differences were observed in the distribution of HR+/HER2+ vs. HR-/HER2+ tumors by race or menopausal status (Table 1).

**Table 1:** Patient demographics and clinicopathological characteristics for Stage I, II, III patients with HER2-positive breast cancer

*At time of diagnosis of index cancer*				
**Variable** **N (%)**	**Total** (*N *= 3394)	**HR-positive** (*n *= 2015)	**HR-negative** (*n *= 1379)	***P*-value**

**Age, mean (se), y **	52.48 (0.21)	52.41 (0.29)	52.59 (0.32)	

**Age category, N, (%)**				<.001

*0 to <50*	1,514 (45)	931 (46)	583 (42)	
*50 to <70*	1,547 (46)	865 (43)	682 (49)	
*70+*	333 (10)	219 (11)	114 (8)	

**Race/Ethnicity, N, (%)**				0.030

*African-American *	259 (8)	135 (7)	124 (9)	
*Caucasian *	2,693 (79)	1,627 (81)	1,066 (77)	
*Hispanic*	262 (8)	155 (8)	107 (8)	
*Asian Pacific Island*	126 (4)	64 (3)	62 (5)	
*American Indian *	10 (<1)	4 (<1)	6 (<1)	
*Other *	16 (<1)	10 (1)	6 (<1)	
*Unknown*	28 (1)	20 (1)	8 (1)	

**Menopausal Status, N, (%)**				0.001

*Premenopausal*	1,569 (46)	978 (49)	591 (43)	
*Postmenopausal*	1,825 (54)	1,037 (51)	788 (57)	

**Body Mass Index kg/m2, N, (%)**				0.512

*<18.5 kg/m2 (Underweight)*	52 (2)	34 (2)	18 (1)	
*18.5 to <25 kg/m2 (Normal)*	1,263 (37)	750 (37)	513 (37)	
*25 to <30 kg/m2 (Obese)*	913 (27)	554 (27)	359 (26)	
*>30 kg/m2 (Severely Obese)*	884 (26)	506 (25)	378 (27)	
*Missing/unknown*	282 (8)	171 (8)	111 (8)	

**Co-morbidity score, N, (%)**				0.958

*0*	2,713 (80)	1,609 (80)	1,104 (80)	
*1*	452 (13)	271 (13)	181 (13)	
*2+*	229 (7)	135 (7)	94 (7)	

**Method of detection, N, (%)**				<.001

*Abnormal screening mammogram*	1,209 (36)	755 (37)	454 (33)	
*Symptom*	2,012 (59)	1,175 (58)	837 (61)	
*Other*	147 (4)	67 (3)	80 (6)	
*Unknown*	26 (1)	18 (1)	8 (1)	

**T stage, category, N, (%)**				<.001

*T1*	1,753 (52)	1,103 (55)	650 (47)	
*T2*	1,150 (34)	691 (34)	459 (33)	
*T3*	244 (7)	129 (6)	115 (8)	
*T4*	197 (6)	73 (4)	124 (9)	
*Unknown*	50 (1)	19 (1)	31 (2)	

**Nodal status, N, (%)**				<.001

*Positive*	1,623 (48)	907 (45)	716 (52)	
*Negative*	1,757 (52)	1,101 (55)	656 (48)	
*Unknown*	14 (<1)	7 (<1)	7 (<1)	

**AJCC stage, N, (%)**				<.001

*I*	1,187 (35)	772 (38)	415 (30)	
*II*	1,467 (43)	887 (44)	580 (42)	
*III*	740 (22)	356 (18)	384 (28)	

**Histologic grade, N, (%)**				<.001

*Low/Intermediate*	907 (27)	719 (36)	188 (14)	
*High*	2,324 (68)	1,211 (60)	1,113 (81)	
*Unknown*	163 (5)	85 (4)	78 (6)	

**Histology, N, (%)**				<.001

*Invasive ductal*	3,101 (91)	1,772 (88)	1,329 (97)	
*Invasive lobular*	105 (3)	89 (4)	16 (1)	
*Mixed ductal and lobular*	139 (4)	120 (6)	19 (1)	
*Other *	49 (1)	34 (2)	15 (1)	

**LVI, N, (%)**				0.630

*Presence*	1,164 (34)	678 (34)	486 (35)	
*Absence*	2,113 (62)	1,267 (63)	846 (61)	
*Unknown*	117 (3)	70 (3)	47 (3)	

**EIC, N, (%)**				0.536

*Presence *	606 (18)	353 (18)	253 (18)	
*Absence*	2,788 (82)	1,662 (82)	1,126 (82)	

***In patients with documented recurrence***				

**Variable**	**Total** *N *= 458	**HR-positive** *N *= 208	**HR-negative** *N *= 250	***P*-value**

*Disease free interval median (range), y*	1.95 (7.69)	2.24 (7.69)	1.70 (7.08)	
*Age, mean (se), y *	50.95 (0.60)	50.39 (0.94)	51.41 (0.78)	

**Race/Ethnicity, N, (%)**				0.644

*African-American *	51 (11)	21 (10)	30 (12)	
*Caucasian *	358 (78)	166 (80)	192 (77)	
*Hispanic*	31 (7)	12 (6)	19 (8)	
*Asian Pacific Island*	12 (3)	6 (3)	6 (2)	
*American Indian *	1 (<1)	1 (<1)	0 (0)	
*Other *	4 (1)	1 (<1)	3 (1)	
*Unknown*	1 (<1)	1 (<1)	0 (0)	

**Menopausal status at time of diagnosis of index cancer, N, (%)**				0.207

*Premenopausal*	223 (49)	108 (52)	115 (46)	
*Postmenopausal*	235 (51)	100 (48)	135 (54)	

**Co-morbidity score at time of diagnosis of index cancer, N, (%)**				0.437

*0*	371 (81)	166 (80)	205 (82)	
*1*	57 (12)	25 (12)	32 (13)	
*2+*	30 (7)	17 (8)	13 (5)	

**AJCC stage at time of diagnosis of index cancer, N, (%)**				0.437

*I*	77 (17)	36 (17)	41 (16)	
*II*	188 (41)	91 (44)	97 (39)	
*III*	193 (42)	81 (39)	112 (45)	

**Histologic grade at time of diagnosis of index cancer, N, (%)**				0.016

*Low/Intermediate*	65 (14)	40 (19)	25 (10)	
*High*	379 (83)	163 (79)	216 (86)	
*Unknown*	14 (3)	5 (2)	9 (4)	

*Note: *AJCC, American Joint Committee on Cancer Staging; EIC, extensive intraductal component; HR, Hormone receptor; LVI, Lymphovascular invasion; se, standard error; y, year

Most (79%) patients received neoadjuvant or adjuvant chemotherapy. Overall, 44% of patients received neoadjuvant or adjuvant trastuzumab. As expected, receipt of adjuvant or neoadjuvant trastuzumab increased over time (8% of patients diagnosed in 2000, 66% of patients diagnosed in 2005, and 77% of patients diagnosed in 2007, data not shown) (Additional file 1, Table S1).

Recurrence was documented in 458 women. Of these, 45% had HR+/HER2+ tumors and 55% had HR-/HER2+ tumors. A total of 32 patients experienced recurrences beyond five years (66% HR-positive and 34% HR-negative). At first recurrence, most (68%) of patients received chemotherapy; 86% of these patients received concurrent HER2-based therapy, either trastuzumab or lapatinib (Additional file 1, Table S1).

### Patterns of recurrence

#### Type of first recurrence by HR

At first recurrence, the risks of local vs. distant recurrence by HR status appeared similar (Table 2; Additional file 2, Table S2). Brain involvement was present in 20% of patients with documented recurrence of HR-/HER2+ tumors and in 13% of patients with documented recurrence of HR+/HER2+ tumors (Additional file 3, Table S3). Bone disease was present in 28% of patients with HR+/HER2+ breast cancer and in 17% of patients with HR-/HER2+ tumors (Additional file 3, Table S3). By univariate analysis, patients with HR-negative tumors were more likely to experience brain recurrence (OR = 1.75, 95% CI: 1.05 to 2.93, *P *= 0.033), and less likely to have bone recurrence (OR = 0.53, 95% CI: 0.34 to 0.82, *P *= 0.005). After multivariable adjustment, the difference in risk of bone recurrence persisted (OR = 0.53, 95% CI: 0.34 to 0.83, *P *= 0.005); however, the difference in risk of brain recurrence was only borderline significant (OR = 1.63, 95% CI: 0.95 to 2.81, *P *= 0.079). No differences in risk of lung or liver as the first site of recurrence by HR were observed (Table 2). The type of recurrence among patients with documented recurrence in the early and late recurring subgroup is represented in Additional files 4, 5, 6, 7, Tables S4-7. Overall the pattern of recurrence in the early recurring subgroup is similar to the overall cohort. In the late recurring subgroup, bone disease was present in 19% of patients with HR+/HER2+ tumors and in 9% of patients with HR-/HER2+ tumors. Brain involvement was present in 24% with HR+/HER2+ tumors and in 18% of patients with HR-/HER2+ tumors. Lung disease was present in 38% of patients with late recurrence versus 15% in the early recurring subgroup.

**Table 2:** Results of logistic regression for site of first (s) of recurrence

	Univariate analysis OR (95% CI)	*P*-value	Multivariable analysis OR (95% CI)	*P*-value
	**HR-negative vs**. **HR-positive**		**HR-negative vs**. **HR-positive**	

**Local regional** **vs. Distant**	1.06 (0.71, 1.59)	0.772	1.15 (0.75, 1.76)	0.531
**Brain vs. Other **	1.75 (1.05, 2.93)	0.033	1.63 (0.95, 2.81)	0.079
**Bone vs. Other**	0.53 (0.34, 0.82)	0.005	0.53 (0.34, 0.83)	0.005
**Lung vs. Other**	1.24 (0.76, 2.03)	0.393	1.27 (0.77, 2.09)	0.353
**Liver vs. Other**	1.38 (0.86, 2.22)	0.188	1.44 (0.89, 2.35)	0.139

Note: HR, Hormone receptor. Adjusting by age at diagnosis, stage and adjuvant trastuzumab.

#### Type of first and subsequent recurrence by HR

Combining first and subsequent sites of recurrence, brain involvement was present in 40% of patients with documented recurrence of HR-/HER2+ breast cancer and in 33% of patients with documented recurrence of HR+/HER2+ tumors. Thirty-four percent of patients with HR-/HER2+ tumors had bone recurrence compared to 49% of patients with HR+/HER2+ tumors. Lung involvement was present in 40% of patients with HR-/HER2+ tumors and in 31% of patients with HR+/HER2+ tumors. As such, combining first and subsequent sites of recurrence, patients with HR-negative tumors were slightly more likely to experience lung involvement (OR = 1.48, 95% CI: 1.00 to 2.18, *P *= 0.05) and less likely to recur in bone (OR = 0.54, 95% CI: 0.37 to 0.78, *p*= 0.001). In multivariable analysis, this pattern persisted for bone recurrence (OR = 0.55, 95% CI: 0.37 to 0.80, *P *= 0.002) and became even less significant for lung recurrence (OR = 1.45, 95% CI: 0.98 to 2.15, *P *= 0.061). No association of HR with brain or liver disease was found. In the multivariable model, young age (OR = 1.66, 95% CI: 1.11 to 2.47, *P *= 0.014), higher stage at presentation (Stage III vs. Stage I: OR = 2.05, 95% CI: 1.12 to 3.75, *P *= 0.020) and adjuvant trastuzumab use (OR = 1.61, 95% CI: 1.06 to 2.46, *P *= 0.025) were significantly associated with brain involvement when compared to other sites of recurrence. Adjuvant trastuzumab was associated with a decrease in the risk of liver recurrence vs. other sites of recurrence (OR = 0.63, 95% CI: 0.41 to 0.96, *P *= 0.033). No other differences in recurrence sites with adjuvant trastuzumab were observed (Table 3; Additional files 8, 9, Tables S8, S9).

**Table 3:** Results of logistic regression for site of first (s) and subsequent of recurrence

	Univariate analysis OR (95% CI)	*P*-value	Multivariate analysis OR (95% CI)	*P*-value
	**HR-negative vs. positive**		**HR-negative vs. positive**	

**Local regional** **vs**. **Distant**	0.71 (0.44,1.15)	0.162	0.74 (0.44, 1.23)	0.246
**Brain vs. Other**	1.37 (0.93, 2.02)	0.107	1.31 (0.88, 1.95)	0.191
**Bone vs. Other**	0.54 (0.37, 0.78)	0.001	0.55 (0.37, 0.80)	0.002
**Lung vs. Other**	1.48 (1.00, 2.18)	0.050	1.45 (0.98, 2.15)	0.061
**Liver vs. Other**	1.01 (0.69, 1.48)	0.954	1.06 (0.72, 1.56)	0.764

Note: HR, Hormone receptor. Adjusting by age at diagnosis, stage and adjuvant trastuzumab.

### Agreement between primary and metastatic samples

Among patients with available data (n = 126 for evaluation of ER status, n = 123 for evaluation of PR status, and n = 121 for evaluation of HER2 status), ER status changed (positive to negative, or vice versa) in 22% of patients; PR status changed (positive to negative, or vice versa) in 29% of patients; HER2 status changed from positive to negative in 17% of patients. Because the cohort was defined as HER2-positive patients, we were unable to describe the number of patients whose HER2 converted from negative to positive. There was a 49% rate of any discordance within the available data (Table 4).

**Table 4:** Agreement between HR and HER2 status from primary and metastatic samples

	HR status*				HER2*	
	**ER status**	**K** **(ASE)**	**PR status**	**K** **(ASE)**	**HER2 Status**	**K** **(ASE)**

**Total (N)**	126		123		121	
**Agreement**, **N (%)**						

*Yes*	98 (78)	0.55 (0.074)	86 (70)	0.31 (0.083)	101 (83)	0.003 (0.019)
*No*	28 (22)			37 (29)		20 (17)
* + to - *	18 (14)			30 (24)		20 (17)
* - to +*	10 (8)			7 (6)		N/A

Note: + to - , change from positive to negative marker; - to +, change from negative to positive marker; ER, estrogen receptor; HER2, Human epidermal growth factor 2; HR, Hormone receptor; PR, progesterone receptor. *Data were available in a limited number of samples due to unknown information in the metastatic setting.

### Survival outcomes

**Figure 1:**
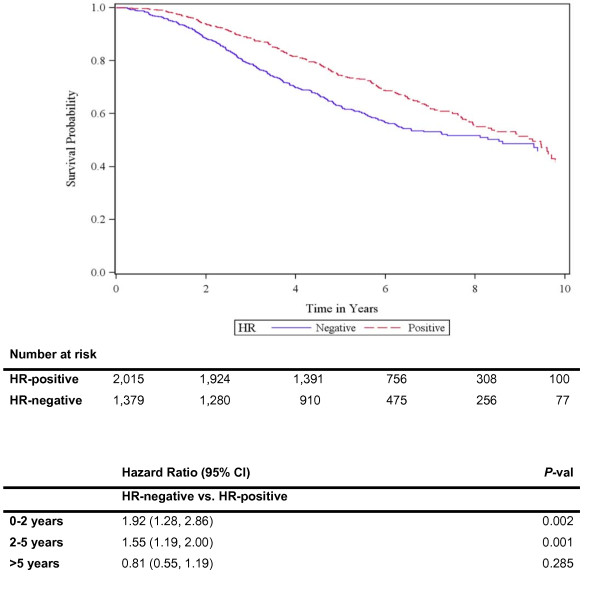
**Overall survival since time of diagnosis by HR status**. Note: HR, Hormone receptor. Adjusted by age, race/ethnicity, stage at diagnosis, grade and year of diagnosis.

Among 3,394 patients, death was recorded in 13% of patients (N = 457), and breast-cancer specific death in 10% of patients (N = 337). The risk of death was not proportional over time for HR status and time dependent covariates yielded patients with HR-negative tumors having significantly increased hazard in the first five years (hazard ratio of death zero to two years = 1.92, 95% CI: 1.28 to 2.86, *P *= 0.002; hazard ratio of death two to five years = 1.55, 95% CI: 1.19 to 2.00, *P *= 0.001; hazard ratio of death more than five years = 0.81, 95% CI: 0.55 to 1.19, *P *= 0.285) (Figure 1). Similar results were seen for BCSS (data not shown).

**Figure 2:**
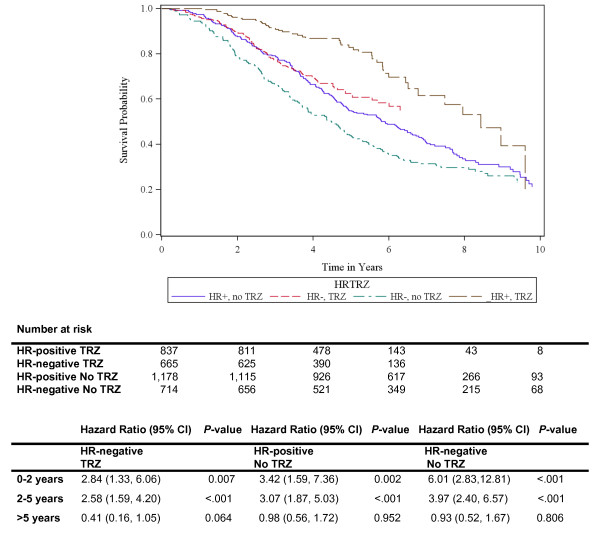
**Overall survival since time of diagnosis by HR status and receipt adjuvant trastuzumab**. Note: Reference group are those who are HR positive and received Trastuzumab. HR, Hormone receptor; TRZ, Trastuzumab. Adjusted by age, race/ethnicity, stage at diagnosis, grade and year of diagnosis.

HR-negative status and lack of adjuvant trastuzumab were associated with increased hazards. The hazards for patients with HR-negative tumors who did not receive adjuvant trastuzumab (median survival for HR-negative tumors that did not receive trastuzumab: 4.48 years) were amplified when compared to patients with HR-positive tumors who received trastuzumab (median survival for HR-positive tumors that received trastuzumab: 8.43 years) (Figure 2).

## Discussion

In this large cohort of patients with Stage I to III HER2-positive breast cancer, we found significant associations between HR status and presenting features, patterns of recurrence and survival outcomes. As compared with patients with HR-positive tumors, those with HR-negative tumors were less likely to recur in bone. In addition, 20% of patients with HR-negative tumors recurred first in the brain compared with 13% of HR-positive patients. Moreover, HR-negative patients experienced more deaths in the first five years, compared with HR-positive patients, but no statistically significant differences in hazards of death beyond Year 5 were found.

Our studies confirm other studies regarding the impact of HR status on sites of recurrence in HER2 disease [13,21-23]. Non-overlapping organ-specific signatures of recurrence in breast cancer have previously been recognized. Moreover, molecular subtypes have been associated with preferential sites of relapse. For instance, in the luminal subtypes (HR-positive subtypes) bone relapse is frequent and brain relapse is usually less common [24-29]. In our study, this pattern of recurrence was also found in the HR+/HER2+ tumors. Our results suggest that HR status remains a major driver of clinical phenotype, even among HER2-positive patients.

Due to the small number of patients with late recurrence, we cannot draw definitive conclusions about recurrence patterns by HR in patients with early versus late recurrences and, therefore, our results are only hypothesis-generating. A total of 38% of patients in the late recurring subgroup relapsed in the lung, with no apparent difference according to HR status; whereas 15% of patients in the early recurring group had recurrence in the lung (including 13% of HR-positive patients and 18% of HR-negative patients). In the early recurrence subgroup, HR-negative patients seemed to be more likely to experience brain relapse as the first site of recurrence compared with HR-positive patients. This difference appears to disappear in the late recurring subgroup. It would be of potential interest to explore other datasets to determine if these findings are consistent across studies.

Overall, the incidence of brain metastasis in HER2-positive disease has been described as 25 to 55% [30-33]. Some, but not all, studies have reported differences in the frequency of brain metastases according to HR status. Kennecke *et al*. reported a frequency of 29% in HR-/HER2+ disease compared with 15% in HR+/HER2+ disease [13]. Brufsky *et al*. reported frequencies of 44% and 31%, respectively, among patients with either HR-/HER2+ disease, compared with HR+/HER2+ disease [33].

In the adjuvant trastuzumab trials in HER2-positive disease, the overall risk of brain recurrence was small (NSABP B-31/NCCTG N9831 trials: 1%, HERA trial: 1%); however, the brain represented an important proportion of the sites for first recurrences (NSABP B-31/NCCTG N9831 trial: 18%, HERA trial: 10%) [34,35].

Similarly, in our study, 20% of first recurrences in patients with HR-negative tumors and 13% of first recurrences in HR-positive patients occurred in the brain. Combining first and subsequent sites of recurrence, 40% of HR-negative patients and 33% of HR-positive patients had brain involvement. The most striking differences were found in the incidence of the brain as the site of first recurrence, with differences between the groups attenuating over time and after adjusting to clinicopathological factors. Similar to previous findings, young age, higher presenting stage and adjuvant trastuzumab use were associated with a higher likelihood of brain recurrence [36].

It is notable that previous efforts to define a gene signature predictive of central nervous system (CNS) relapse among patients with HER2-positive disease did not succeed in identifying a predictor of the presence or absence of CNS relapse, but were successful in distinguishing between those patients with early versus late brain relapse [37]. Our data support the hypothesis that biologic factors predisposing to early brain relapse are probably not the same as those predisposing to later CNS involvement. Moreover, within the subgroup of patients with early relapses, 20% of HR-negative patients had the brain as the first site of recurrence versus 11% of HR-positive patients; within the subgroup of patients with late relapses, 18% of patients had the brain as the first site of recurrence versus 24% of HR-positive patients.

Multiple prior studies have described differences in the hazard of relapse and death over time according to HR status [20,38]. We found that HR status is also associated with the hazard of death over time within the HER2-positive population. Patients with HR-/HER2+ tumors faced a higher risk of death within five years of initial diagnosis; however, beyond five years no statistically significant differences in hazards of death between groups were found. Similar results can be observed when observing the shape of the survival curves in the adjuvant trials. For example, both in the CALGB9344/INT0148 and in the HERA trial this difference in the hazard of relapse over time is apparent [12,15].

Concordant with previous knowledge, trastuzumab increases the rates of survival independently of HR status. After stratifying by adjuvant trastuzumab use, the differences in survival by HR status persisted. It is worth noting that despite a lower rate of pathologic complete responses (pCR) among patients with HR+/HER2+ tumors across neoadjuvant trials [10,39-45], the overall prognosis of such patients is not worse than those with HR-/HER2+ tumors. These findings also suggest that in patients with HR+/HER2+ tumors, lack of pCR will, potentially, have less prognostic value than among patients with HR-/HER2+ tumors [46].

Finally, we explored the agreement in ER, PR and HER2 between primary and metastatic biopsies. Previous studies showed that discordance rates between primary and metastatic samples range between 10% and 40%. Discordance may be associated with: a) methodological factors, b) intratumoral heterogeneity and c) clonal selection associated with adjuvant therapy. The relative contribution of each of these factors is not determined, although it is believed that a true switch in biology should be a rare event [47]. We found a 49% rate of any discordance between primary and metastatic samples, including a switch from HER2-positive to HER2-negative in 17% of cases. The influence of neoadjuvant chemotherapy plus trastuzumab on HER2 has been investigated, with a loss of HER2 amplification/overexpression in 12% to 43% of the cases with residual disease [48]. Previous studies suggested that discordance between primary and metastatic samples can have prognostic value [49,50], and further investigation of the biologic, prognostic and predictive value of the discordance between primary and metastatic samples is warranted.

Our study had a number of strengths. We had access to a large prospectively collected dataset which drew from large centers distributed geographically across the U.S. Because the sample was limited to patients who initially presented with early stage disease, we could adjust for clinically relevant tumor- and treatment-related variables. Unlike the majority of adjuvant trials, we captured all sites of both first and subsequent recurrence, and thus were able to provide a broader picture of the natural history of HER2-positive disease. In addition, analysis of metastatic patterns in our study was less biased than if we had relied on clinical trials in the metastatic setting, where inclusion/exclusion criteria could introduce significant selection bias (for example, restrictions on patients with brain metastases, elevated liver function tests, and so on). Finally, by only including patients with metastatic disease who were initially seen at time of early stage diagnosis, we hoped to minimize potential referral bias, which is a valid concern given the nature of the NCCN participating institutions. For instance, patients referred to an NCCN center for the first time to consider a third-line chemotherapy trial would not be included in this dataset.

Our study also had several limitations. HR status was based on the assessment provided by the breast cancer pathologists at the time of diagnosis. We did not conduct central pathologic review of the tumor samples nor did we require uniform pre-specified cut-offs for these markers. The classification was based on the standards used in each center applicable at the time of diagnosis. It is likely that academic pathologists at the participating NCCN institutions reviewed specimens as part of routine clinical care, which is reassuring as to the quality of the information provided. However, we acknowledge the potential for some difference in interpretation over time, particularly in the setting of low-level (<10%) expression.

Because the study was limited to patients who presented to NCCN centers, we cannot rule out a potential referral bias. However, it is unlikely that the impact of HR in HER2 disease would be different in a population-based sample, and our data are consistent with other published data of the impact of HR status in HER2-positive disease. Patients received a variety of neoadjuvant and adjuvant therapies and, as expected, the use of trastuzumab increased over time. However, results were not materially different after adjusting for the use of trastuzumab, suggesting that our findings regarding recurrence patterns are relevant to current practice.

With respect to our analysis of discordance in HR and HER2 between primary and metastatic samples, we did not conduct central pathologic review of the tumor samples, and cannot rule out technical issues as a cause of discordance. However, as previously noted, it is likely that almost all specimens were reviewed as part of routine clinical care at the participating NCCN academic institutions.

Finally, we used classical pathological markers (ER/PR and HER2) as a proxy for the intrinsic subtypes. For practical purposes, it is generally accepted that intrinsic subtypes are approximated using clinicopathological criteria [7,51]. However, several groups have reported varying levels of concordance between assignments of subtype by IHC and gene expression array criteria, and most suggest that the information provided by the molecular subtypes expands the information provided by classical clinicopathologic markers. A combined dataset that included 106 patients that belong to the HER2 enriched subtype, comprised 51% ER-negative/HER2-positive, 15% ER-positive/HER2-positive and even 34% HER2-negative analyzed by classical procedures. In a different report, all patients that belong to HER2-like molecular subtype were HER2-positive, 46% ER-positive [7,52]. It is conceivable that using gene expression profiling to classify tumors may have resulted in more dramatic differences in the patterns of recurrence and survival outcomes over time.

## Conclusions

Our study supports the classification of HER2 disease as two different entities distinguished by HR. Further work to understand the relationship between intrinsic subtype and outcomes restricted to patients with clinically HER2-positive disease is warranted. We believe this has implications in both basic and clinical research. In particular, we believe that the next generation of HER2-focused clinical trials should integrate HR status (and/or intrinsic subtype) in their design, either by designing distinct treatment protocols for patients with HR+/HER2+ disease compared to HR-/HER2+ disease, or by allocating patients within strata defined by HR.

## Abbreviations

AJCC: American Joint Committee on Cancer; ASE: asymptotic standard error; BCSS: Breast Cancer Specific Survival; BMI: body mass index; CNS: central nervous system; EIC: extensive intraductal component; ER: estrogen receptor; FISH: Fluorescence *in situ *hybridization; HER-2: Human epidermal growth factor-2; HR: Hormone receptor; HR+/HER2+: HR-positive/HER2-positive; HR-/HER2+HR-negative/HER2-positive; IHC: Immunohistochemistry; LVI: Lymphovascular invasion; NCCN: National Comprehensive Network; NDI: National Death Index; OR: odds ratio; OS: overall survival: pCR: pathologic complete response; PR: progesterone receptor;

## Competing interests

BM is an uncompensated consultant or has an uncompensated advisory role for GlaxoSmithKline and Pfizer. RLT has stock ownership in UpToDate, NUL receives Research Funding from Genentech, GlaxoSmithKline, Infinity Pharmaceuticals, Boehringer Ingelheim, and has a consultant or advisory role for GlaxoSmithKline (compensated), Novartis (compensated) and Genentech (uncompensated). IVL, RAO, MEH, PKM, HSR, JW, JCN and JCW declare that they have no competing interests.

## Authors' contributions

IVL and NUL conceived the study and the design of the study, participated in the analysis and interpretation of the data, and coordinated and drafted the manuscript. RAO participated in the design of the study, performed the statistical analysis, participated in the interpretation of the data and helped in drafting the manuscript. MEH participated in the design of the study, statistical analysis, interpretation of the data, coordination and helped in drafting the manuscript. JCW participated in the design of the study, interpretation of the data, coordination, and helped in drafting the manuscript. PKM, BM, HSR, RLT, JW and JCN participated in the interpretation of the data and helped in drafting the manuscript. All authors read and approved the final manuscript.

## Supplementary Material

Additional file 1 **Table S1. Treatment characteristics**. Treatment characteristics at the time of diagnosis and at the time of first recurrence. Note: HR, Hormone receptor. Click here for file

Additional file 2 **Table S2. Type of first (s) recurrence by HR among patients with documented recurrence - type of site of first(s) recurrence date**. Type of site of first(s) recurrence (local/regional, distant, combined) by HR among patients with documented recurrence. Click here for file

Additional file 3 **Table S3. Type of first (s) recurrence by HR among patients with documented recurrence - type of site diagnosed on first recurrence(s) date**. Type of site of first(s) recurrence (ipsilateral breast, chest wall/local nodes/regional nodes, contralateral breast, bone, lung, liver, brain, all other sites) by HR among patients with documented recurrence. * Analysis based on cohort of 458 patients (208, HR positive; 250, HR negative) with documented recurrence, representing a total of 553 sites of recurrence. Proportion of patients does not add up to 100% as patients could have more than one site of recurrence. Click here for file

Additional file 4 **Table S4. Type of first (s) recurrences by HR among patients with documented recurrence - type of first(s) recurrences in the early recurring subgroup**. Type of site of first(s) recurrence (local/regional, distant, combined) by HR among patients with documented early recurrence. Click here for file

Additional file 5 **Table S5. Type of first (s) recurrences by HR among patients with documented recurrence - type of site diagnosed on first(s) recurrences in the early recurring subgroup**. Type of site of first(s) recurrence (ipsilateral breast, chest wall/local nodes/regional nodes, contralateral breast, bone, lung, liver, brain, all other sites) by HR among patients with documented early recurrence.* Analysis based on cohort of 426 patients (187, HR positive; 239, HR negative) with documented recurrence, representing a total of 515 sites of recurrence. Proportion of patients does not add up to 100% as patients could have more than one site of recurrence Click here for file

Additional file 6 **Table S6. Type of first (s) recurrences by HR among patients with documented recurrence - type of first(s) recurrences in the late recurring subgroup**. Type of site of first(s) recurrence (local/regional, distant, combined) by HR among patients with documented late recurrence. Click here for file

Additional file 7 **Table S7. Type of first (s) recurrences by HR among patients with documented recurrence - type of site diagnosed on first(s) recurrences in the late recurring subgroup**. Type of site of first(s) recurrence (ipsilateral breast, chest wall/local nodes/regional nodes, contralateral breast, bone, lung, liver, brain, all other sites) by HR among patients with documented late recurrence. *Analysis based on cohort of 32 patients (21, HR positive; 11, HR negative) with documented recurrence, representing a total of 38 sites of recurrence. Proportion of patients does not add up to 100% as patients could have more than one site of recurrence. Click here for file

Additional file 8 **Table S8. Type of first (s) and subsequent recurrences by HR among patients with documented recurrence-type of first(s) and subsequent recurrences**. Type of site of first(s) and subsequent recurrence (local/regional, distant, combined) by HR among patients with documented recurrence. Click here for file

Additional file 9 **Table S9. Type of first (s) and subsequent recurrences by HR among patients with documented recurrence-type of site diagnosed on first(s) and subsequent recurrences**. Type of site of first(s) recurrence (ipsilateral breast, chest wall/local nodes/regional nodes, contralateral breast, bone, lung, liver, brain, all other sites) by HR among patients with documented recurrence. *Analysis based on cohort of 458 patients (208, HR positive; 250, HR negative) with documented recurrence, representing a total of 1,014 sites of recurrence. Proportion of patients does not add up to 100% as patients could have more than one site of recurrence. Click here for file
