# Supplementary material for: Impact of hormone receptor status on patterns of recurrence and clinical outcomes among patients with human epidermal growth factor-2-positive breast cancer in the National Comprehensive Cancer Network: a prospective cohort study
Source: Breast Cancer Res. 2012 Oct 1;14(5):R129. doi: 10.1186/bcr3324 (PMC4053106; doi:10.1186/bcr3324)
Supplement: Additional file 1 — Table S1. Treatment characteristics. Treatment characteristics at the time of diagnosis and at the time of first recurrence. Note: HR, Hormone receptor. [file bcr3324-S1.PDF]

At time of diagnosis			
**Variable N (%)**	**Total** (*N*=3394)	**HR-positive** (*n*=2015)	**HR-negative** (*n*= 1379)
**Neo or adjuvant chemotherapy, N (%)**			
No	712 (21)	502 (25)	210 (15)
Yes	2682 (79)	1513 (75)	1169 (85)
Anthracycline-containing regimen	587 (22)	380 (25)	207 (18)
Anthracycline-containing regimen-taxane,	1820 (68)	973 (64)	847 (72)
Other	275 (10)	160 (11)	115 (10)
**Neo or adjuvant trastuzumab, N (%)**			
No	1892 (56)	1178 (58)	714 (52)
Yes	1502 (44)	837 (42)	665 (48)
**Adjuvant hormonal therapy, N (%)**			
No	1643 (48)	318 (16)	1325 (96)
Yes	1751 (52)	1697 (84)	54 (4)
At time of first recurrence			
**Variable N (%)**	**Total** (*N*=458)	**HR-positive** (*n*=208)	**HR-negative** (*n*=250)
**Chemotherapy, N (%)**			
No	148 (32)	73 (35)	75 (30)
Yes	310 (68)	135 (65)	175 (70)
**Trastuzumab, N (%)**			
No	167 (36)	77 (37)	90 (36)
Yes	291 (64)	131 (63)	160 (64)
**Lapatanib, N (%)**			
No	358 (78)	165 (79)	193 (77)
Yes	100 (22)	43 (21)	57 (23)
**Hormonal therapy, N (%)**			
No	337 (74)	98 (47)	239 (96)
Yes	121 (26)	110 (53)	11 (4)
**Enrollment on clinical trial, N (%)**			
No	385 (84)	172 (83)	213 (85)
Yes	73 (16)	36 (17)	37 (15)
