# Supplementary material for: Impact of hormone receptor status on patterns of recurrence and clinical outcomes among patients with human epidermal growth factor-2-positive breast cancer in the National Comprehensive Cancer Network: a prospective cohort study
Source: Breast Cancer Res. 2012 Oct 1;14(5):R129. doi: 10.1186/bcr3324 (PMC4053106; doi:10.1186/bcr3324)
Supplement: Additional file 2 — Table S2. Type of first (s) recurrence by HR among patients with documented recurrence - type of site of first(s) recurrence date. Type of site of first(s) recurrence (local/regional, distant, combined) by HR among patients with documented recurrence. [file bcr3324-S2.PDF]

N (%)	**Total** (*N*=458)	**HR-positive** (*n*=208)	**HR-negative** (*n*=250)
**Local/Regional**	133 (29)	59 (28)	74 (30)
**Distant**	314 (69)	144 (69)	170 (68)
**Combined**	11 (2)	5 (2)	6 (2)
