# Supplementary material for: Impact of hormone receptor status on patterns of recurrence and clinical outcomes among patients with human epidermal growth factor-2-positive breast cancer in the National Comprehensive Cancer Network: a prospective cohort study
Source: Breast Cancer Res. 2012 Oct 1;14(5):R129. doi: 10.1186/bcr3324 (PMC4053106; doi:10.1186/bcr3324)
Supplement: Additional file 3 — Table S3. Type of first (s) recurrence by HR among patients with documented recurrence - type of site diagnosed on first recurrence(s) date. Type of site of first(s) recurrence (ipsilateral breast, chest wall/local nodes/regional nodes, contralateral breast, bone, lung, liver, brain, all other sites) by HR among patients with documented recurrence. * Analysis based on cohort of 458 patients (208, HR positive; 250, HR negative) with documented recurrence, representing a total of 553 sites of recurrence. Proportion of patients does not add up to 100% as patients could have more than one site of recurrence. [file bcr3324-S3.PDF]

N (%)*	**Total** (*N*=458)	**HR-positive** (*n*=208)	**HR-negative** (*n*=250)
**Ipsilateral breast**	84 (18)	35 (17)	49 (20)
**Chest wall, local/regional nodes**	61 (13)	29 (14)	32 (13)
**Contralateral breast**	2 (<1)	1 (<1)	1 (<1)
**Bone**	102 (22)	59 (28)	43 (17)
**Lung**	78 (17)	32 (15)	46 (18)
**Liver**	87 (19)	34 (16)	53 (21)
**Brain**	76 (17)	26 (13)	50 (20)
**All other sites**	63 (14)	23 (11)	40 (16)
