# Supplementary material for: Impact of hormone receptor status on patterns of recurrence and clinical outcomes among patients with human epidermal growth factor-2-positive breast cancer in the National Comprehensive Cancer Network: a prospective cohort study
Source: Breast Cancer Res. 2012 Oct 1;14(5):R129. doi: 10.1186/bcr3324 (PMC4053106; doi:10.1186/bcr3324)
Supplement: Additional file 4 — Table S4. Type of first (s) recurrences by HR among patients with documented recurrence - type of first(s) recurrences in the early recurring subgroup. Type of site of first(s) recurrence (local/regional, distant, combined) by HR among patients with documented early recurrence. [file bcr3324-S4.PDF]

N (%)	**Total** (*N*=426)	**HR-positive** (*n*=187)	**HR-negative** (*n*=239)
**Local/Regional**	125 (29)	55 (29)	70 (29)
**Distant**	290 (68)	127 (68)	163 (68)
**Combined**	11 (3)	5 (3)	6 (3)
