# Supplementary material for: Impact of hormone receptor status on patterns of recurrence and clinical outcomes among patients with human epidermal growth factor-2-positive breast cancer in the National Comprehensive Cancer Network: a prospective cohort study
Source: Breast Cancer Res. 2012 Oct 1;14(5):R129. doi: 10.1186/bcr3324 (PMC4053106; doi:10.1186/bcr3324)
Supplement: Additional file 5 — Table S5. Type of first (s) recurrences by HR among patients with documented recurrence - type of site diagnosed on first(s) recurrences in the early recurring subgroup. Type of site of first(s) recurrence (ipsilateral breast, chest wall/local nodes/regional nodes, contralateral breast, bone, lung, liver, brain, all other sites) by HR among patients with documented early recurrence.* Analysis based on cohort of 426 patients (187, HR positive; 239, HR negative) with documented recurrence, representing a total of 515 sites of recurrence. Proportion of patients does not add up to 100% as patients could have more than one site of recurrence [file bcr3324-S5.PDF]

N (%)*	Total (*N*=426)	HR-positive (*n*=187)	HR-negative (*n*=239)
**Ipsilateral breast**	80 (19)	34 (18)	46 (19)
**Chest wall, local/regional nodes**	57 (13)	26 (14)	31 (13)
**Contralateral breast**	2 (<1)	1 (<1)	1 (<1)
**Bone**	97 (23)	55 (29)	42 (18)
**Lung**	66 (15)	24 (13)	42 (18)
**Liver**	84 (20)	34 (18)	50 (21)
**Brain**	69 (16)	21 (11)	48 (20)
**All other sites**	60 (14)	20 (11)	40 (17)
