# Supplementary material for: Impact of hormone receptor status on patterns of recurrence and clinical outcomes among patients with human epidermal growth factor-2-positive breast cancer in the National Comprehensive Cancer Network: a prospective cohort study
Source: Breast Cancer Res. 2012 Oct 1;14(5):R129. doi: 10.1186/bcr3324 (PMC4053106; doi:10.1186/bcr3324)
Supplement: Additional file 6 — Table S6. Type of first (s) recurrences by HR among patients with documented recurrence - type of first(s) recurrences in the late recurring subgroup. Type of site of first(s) recurrence (local/regional, distant, combined) by HR among patients with documented late recurrence. [file bcr3324-S6.PDF]

N (%)	**Total** (*N*=32)	**HR-positive** (*n*=21)	**HR-negative** (*n*=11)
**Local/Regional**	8 (25)	4 (19)	4 (36)
**Distant**	24 (75)	17 (81)	7 (64)
**Combined**	0 (0)	0 (0)	0 (0)
