# Supplementary material for: Impact of hormone receptor status on patterns of recurrence and clinical outcomes among patients with human epidermal growth factor-2-positive breast cancer in the National Comprehensive Cancer Network: a prospective cohort study
Source: Breast Cancer Res. 2012 Oct 1;14(5):R129. doi: 10.1186/bcr3324 (PMC4053106; doi:10.1186/bcr3324)
Supplement: Additional file 7 — Table S7. Type of first (s) recurrences by HR among patients with documented recurrence - type of site diagnosed on first(s) recurrences in the late recurring subgroup. Type of site of first(s) recurrence (ipsilateral breast, chest wall/local nodes/regional nodes, contralateral breast, bone, lung, liver, brain, all other sites) by HR among patients with documented late recurrence. *Analysis based on cohort of 32 patients (21, HR positive; 11, HR negative) with documented recurrence, representing a total of 38 sites of recurrence. Proportion of patients does not add up to 100% as patients could have more than one site of recurrence. [file bcr3324-S7.PDF]

N (%)*	Total (*N*=32)	HR-positive (*n*=21)	HR-negative (*n*=11)
**Ipsilateral breast**	4 (13)	1 (5)	3 (27)
**Chest wall, local/regional nodes**	4 (13)	3 (14)	1 (9)
**Contralateral breast**	0 (0)	0 (0)	0 (0)
**Bone**	5 (16)	4 (19)	1 (9)
**Lung**	12 (38)	8 (38)	4 (36)
**Liver**	3 (9)	0 (0)	3 (27)
**Brain**	7 (22)	5 (24)	2 (18)
**All other sites**	3 (9)	3 (14)	0 (0)
