# Supplementary material for: Impact of hormone receptor status on patterns of recurrence and clinical outcomes among patients with human epidermal growth factor-2-positive breast cancer in the National Comprehensive Cancer Network: a prospective cohort study
Source: Breast Cancer Res. 2012 Oct 1;14(5):R129. doi: 10.1186/bcr3324 (PMC4053106; doi:10.1186/bcr3324)
Supplement: Additional file 8 — Table S8. Type of first (s) and subsequent recurrences by HR among patients with documented recurrence-type of first(s) and subsequent recurrences. Type of site of first(s) and subsequent recurrence (local/regional, distant, combined) by HR among patients with documented recurrence. [file bcr3324-S8.PDF]

N (%)	**Total** (*N*=458)	**HR-positive** (*n*=208)	**HR-negative** (*n*=250)
**Local/Regional**	80 (18)	42 (20)	38 (15)
**Distant**	283 (62)	130 (63)	153 (61)
**Combined**	95 (21)	36 (17)	59 (24)
