# Supplementary material for: Impact of hormone receptor status on patterns of recurrence and clinical outcomes among patients with human epidermal growth factor-2-positive breast cancer in the National Comprehensive Cancer Network: a prospective cohort study
Source: Breast Cancer Res. 2012 Oct 1;14(5):R129. doi: 10.1186/bcr3324 (PMC4053106; doi:10.1186/bcr3324)
Supplement: Additional file 9 — Table S9. Type of first (s) and subsequent recurrences by HR among patients with documented recurrence-type of site diagnosed on first(s) and subsequent recurrences. Type of site of first(s) recurrence (ipsilateral breast, chest wall/local nodes/regional nodes, contralateral breast, bone, lung, liver, brain, all other sites) by HR among patients with documented recurrence. *Analysis based on cohort of 458 patients (208, HR positive; 250, HR negative) with documented recurrence, representing a total of 1,014 sites of recurrence. Proportion of patients does not add up to 100% as patients could have more than one site of recurrence. [file bcr3324-S9.PDF]

N (%)*	Total (*N*=458)	HR-positive (*n*=208)	HR-negative (*n*=250)
**Ipsilateral breast**	92 (20)	38 (18)	54 (22)
**Chest wall, local/regional nodes**	105 (23)	49 (24)	56 (22)
**Contralateral breast**	8 (2)	5 (2)	3 (1)
**Bone**	185 (40)	101(49)	84 (34)
**Lung**	163 (36)	64 (31)	99 (40)
**Liver**	168 (37)	76 (37)	92 (37)
**Brain**	168 (37)	68 (33)	100 (40)
**All other sites**	125 (27)	49 (24)	76 (30)
